# Force-based assessment of tissue handling skills in simulation training for robot-assisted surgery

**DOI:** 10.1007/s00464-023-09905-y

**Published:** 2023-02-09

**Authors:** A. Masie Rahimi, Sem F. Hardon, E. Willuth, F. Lang, Caelan M. Haney, Eleni A. Felinska, Karl-Friedrich Kowalewski, Beat P. Müller-Stich, Tim Horeman, F. Nickel, Freek Daams

**Affiliations:** 1 Department of Surgery, Amsterdam UMC–VU University Medical Center, Amsterdam, The Netherlands; 2 Amsterdam Skills Centre for Health Sciences, Tafelbergweg 47, 1105 BD Amsterdam, The Netherlands; 3 Cancer Center Amsterdam, Amsterdam, The Netherlands; 4 Department of Biomechanical Engineering, Delft University of Technology, Delft, The Netherlands; 5 Department of General, Visceral and Transplantation Surgery, Heidelberg University, Heidelberg, Germany

**Keywords:** Robotic-assisted surgery, Simulation training, Robotic surgery training, Force measurements, Objective assessment, Robot tissue manipulation

## Abstract

**Introduction:**

Although robotic-assisted surgery is increasingly performed, objective assessment of technical skills is lacking. The aim of this study is to provide validity evidence for objective assessment of technical skills for robotic-assisted surgery.

**Methods:**

An international multicenter study was conducted with participants from the academic hospitals Heidelberg University Hospital (Germany, Heidelberg) and the Amsterdam University Medical Centers (The Netherlands, Amsterdam). Trainees with distinctly different levels of robotic surgery experience were divided into three groups (novice, intermediate, expert) and enrolled in a training curriculum. Each trainee performed six trials of a standardized suturing task using the da Vinci Surgical System. Using the ForceSense system, five force-based parameters were analyzed, for objective assessment of tissue handling skills. Mann–Whitney *U* test and linear regression were used to analyze performance differences and the Wilcoxon signed-rank test to analyze skills progression.

**Results:**

A total of 360 trials, performed by 60 participants, were analyzed. Significant differences between the novices, intermediates and experts were observed regarding the total completion time (41 s vs 29 s vs 22 s *p* = *0.003*), mean non zero force (29 N vs 33 N vs 19 N *p* = 0.032), maximum impulse (40 Ns vs 31 Ns vs 20 Ns *p* = 0.001) and force volume (38 N^3^ vs 32 N^3^ vs 22 N^3^ *p* = 0.018). Furthermore, the experts showed better results in mean non-zero force (22 N vs 13 N *p* = 0.015), maximum impulse (24 Ns vs 17 Ns *p* = 0.043) and force volume (25 N^3^ vs 16 N^3^ *p* = 0.025) compared to the intermediates (*p* ≤ 0.05). Lastly, learning curve improvement was observed for the total task completion time, mean non-zero force, maximum impulse and force volume (*p* ≤ 0.05).

**Conclusion:**

Construct validity for force-based assessment of tissue handling skills in robot-assisted surgery is established. It is advised to incorporate objective assessment and feedback in robot-assisted surgery training programs to determine technical proficiency and, potentially, to prevent tissue trauma.

**Supplementary Information:**

The online version contains supplementary material available at 10.1007/s00464-023-09905-y.

Robotic-assisted surgery (RAS) requires an advanced technical skill set. Due to increased degrees of freedom, the learning curve for RAS is less steep than for laparoscopy and skills that need to be developed are eye-hand coordination, control of the camera and the third arm as primary surgeon, switching between instrument arms, bimanual dexterity, needle handling and suturing, depth perception and tissue manipulation. Therefore, it is important that sufficient time and attention is devoted to achieving and optimizing this skill set for performing RAS [1–3]. Especially as there is no haptic feedback and a reduced tissue feeling. This can be safely obtained by deliberate training in a non-clinical/simulation environment [4].

Previously, our research group validated and analyzed the effect and importance of objective force, motion and time feedback for laparoscopic simulation training [5–7]. There are various modalities for RAS training. The most frequent modality consists of virtual reality training, box training with wet lab (cadaver models) or dry lab (suture pads and biotissue) [8, 9]. The most widely used virtual reality trainer is the da Vinci Skills Simulator (dVSS, Mimic Technologies, Seattle WA, USA) assessing a number of objective parameters: total completion time, instrument collisions, time of excessive force, instruments out of view, economy of motion and master workspace [10]. While VR simulations can be very realistic and provide a good approximation of the tasks and procedures being trained, current simulators cannot naturally replicate the complexity and unpredictability of real-life situations. In particular, for example, the unpredictability and physics of suturing and knot tying.

Box training still often consists of real time or video assessment with subjective forms [11–14]. Despite, the increased risk of unintentional tissue damage due to the absence of haptic feedback in most robotic surgery systems, current training curricula with virtual reality (VR) and box training lack the ability to train and assess tissue manipulation and the application of forces. This complicates training safe tissue manipulation in RAS.

The aim of this study is to obtain construct validity evidence for an objective force and time measuring system in robotic surgery training and to analyze the learning curves.

## Methods

### Study design

In this international multicenter prospective study, participants from two academic hospitals were included: the Dept. of Surgery Heidelberg University Hospital (Germany, Heidelberg) and the Amsterdam University Medical Centers (The Netherlands, Amsterdam).

### Participants

**Fig. 1:**
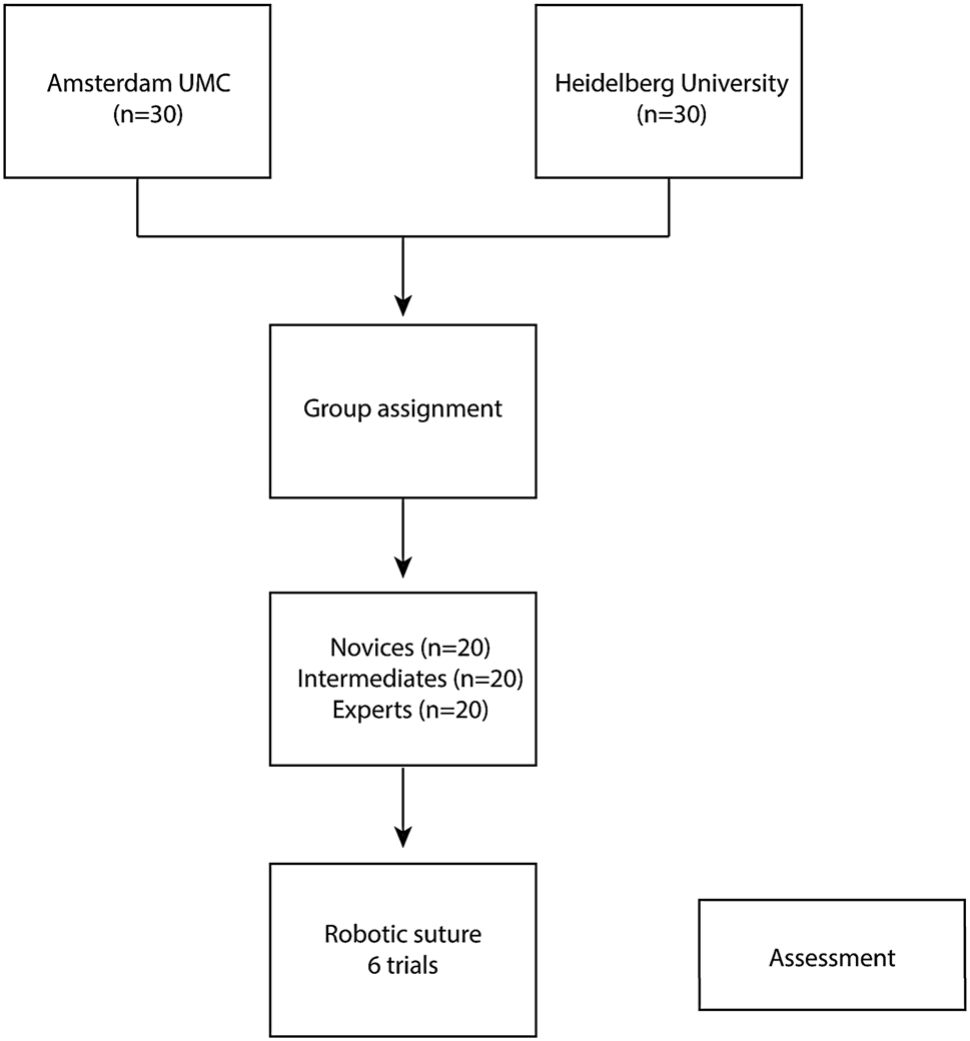
Study design flow chart

Participants were classified and divided into three groups based on their prior robotic surgery experience as operator: novices consisting of junior residents (0 cases and exposure to the system), intermediates consisting of senior residents and young surgeons (< 15 robotic-assisted surgery cases as console surgeon and > 25 robotic knots) and experts consisting of attendees/robot experts (> 15 robotic-assisted surgery cases as console surgeon and > 50 robotic knots) (Fig. 1).

### Systems and hardware

The da Vinci Si Surgical System (Intuitive Surgical Inc., Sunnyvale, California USA) and a box trainer equipped with a suture pad were used for the robotic suturing task (Supplemental File A). The trials were performed using braided multifilament sutures: 12 cm Novosyn 2/0 HR26 (BBraun, Melsungen, Germany). The trials were assessed using the objective ForceSense measuring system (Medishield, Delft, The Netherlands). The ForceSense provided feedback regarding maximum force, mean force, max impulse, force volume and time (Table 1) [6, 15]. All trials were recorded and uploaded to an online database.

**Table 1:** Description of the objective performance metrics of the ForceSense

Parameter	Description
Task time	Total time needed to complete the task presented in seconds (s)
Maximum absolute force (N)	The highest absolute force applied on the training task during tissue manipulation
Mean non zero force (N)	The average force exerted on the training task during tissue manipulation [6, 15]
Max impulse (Ns)	The highest absolute force-over-time integral applied on the training task [6, 15]
Force volume (N^3^)	When viewing the forces in a 3d plane an ellipsoid is imagined. The force volume consists of the multiplication of the forces (and standard deviation) in the height, length and width of the ellipsoid [6, 15]

### Protocol

All trainees received a brief instruction on the da Vinci Surgical System. The trainees were introduced to the technical aspects of the da Vinci Surgical System: ergonomics, user interface, controls, EndoWrist, camera, and clutching. Each trainee performed six separate repetitions of the interrupted robotic surgical suture and knot tying task. Furthermore, the participants received instructions and a video regarding the surgical suture and knot tying task. The suture consisted of a square knot and an additional loop to lock the suture. The camera and the suture were prepared in advance in a standardized fashion and after a countdown, the training task began. This was considered as one trial of the task. All six trials were performed consecutively on the same day.

### Statistical analyses

Data from the online database was analyzed using IBM SPSS statistics 28 (SPSS Inc., Chicago, Illinois USA). Descriptive statistics and frequency measurements were performed to determine the means and standard deviation. GraphPad (Prism 9.0.0, San Diego, California USA) was used for boxplots of the outcomes. Shapiro–Wilk test was performed and the data was not normally distributed. Post hoc power analyses after the initial inclusions was performed (Supplemental file E).

#### Construct validity

A Kruskal–Wallis test was performed to determine whether significant differences were prevalent between the novices, intermediates and experts. Subsequently, Mann–Whitney *U* tests were used to analyze the differences between the groups individually.

To strengthen the construct validation analyses (in addition to comparing outcomes from the different experience groups) a linear regression was also performed. Linear regression analyzed the effect of robotic surgery experience on the different outcome measurements.

#### Learning curve

To determine overall progression of skills and the effectiveness of this short course, the Wilcoxon signed-rank test was conducted to compare the outcomes of the first and the last performed trial. An outcome with a *p* < 0.05 was regarded as statistically significant.

## Results

A total of 360 repetitions, performed by 60 participants, were included for analyses. Nineteen of the participants were female and one of the participants had a dominant left hand.

### Construct validation comparing novices, intermediates and experts

**Fig. 2:**
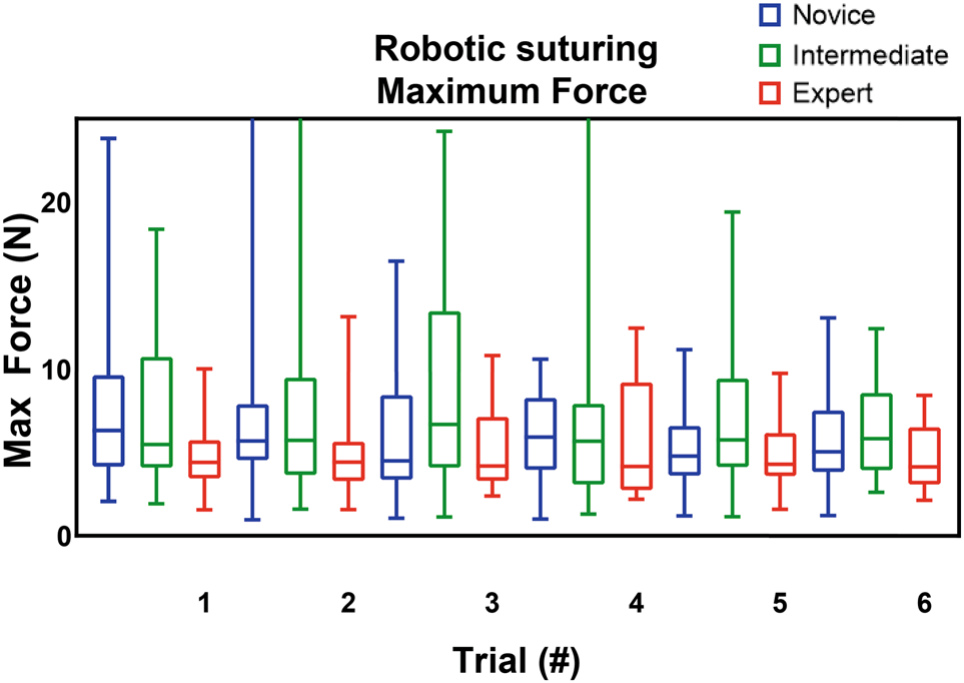
Robotic suturing maximum absolute force (in N)

**Fig. 3:**
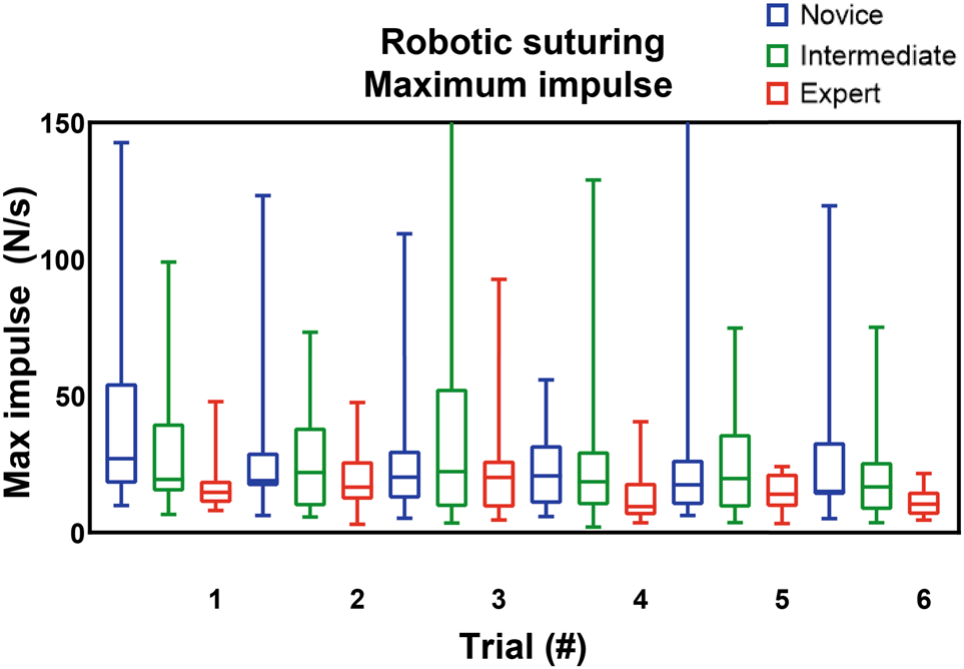
Robotic suturing maximum impulse (in Ns)

**Fig. 4:**
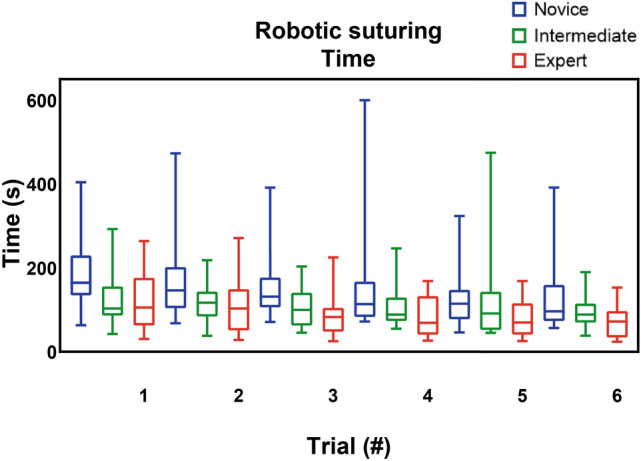
Robotic suturing time (in seconds)

Significant differences between the novices, intermediates and experts were observed regarding the total completion time (41 s vs 29 s vs 22 s *p* = *0.003*), mean non zero force (29 N vs 33 N vs 19 N *p* = 0.032), maximum impulse (40 Ns vs 31 Ns vs 20 Ns *p* = 0.001) and force volume (38 N^3^ vs 32 N^3^ vs 22 N^3^ *p* = 0.018) (Supplemental file B, Table B1). The intermediates and experts had significant better results compared to the novices for all parameters (Table 2 and Figs. 2, 3, 4) (Supplemental file B, Table B2–B3). Furthermore, the experts showed better results in mean non-zero force (22 N vs 13 N *p* = 0.015), maximum impulse (24 Ns vs 17 Ns *p* = 0.043) and force volume (25 N^3^ vs 16 N^3^ *p* = 0.025) compared to the intermediates (*p* ≤ 0.05) (Supplemental file B, Table B4).

**Table 2:** Comparison between the novices and experts for the robotic suturing task. The Mann–Whitney *U* test was performed to determine any significant differences between the groups

	Novice (*n* = 20)	Intermediate (*n* = 20)	*Z*-value	*p*
Total time (s)				
Trial 1	25.10	15.90	− 2.489	**0.012**
Trial 2	23.70	17.30	− 1.731	0.086
Trial 3	24.65	16.35	− 2.245	**0.024**
Trial 4	23.70	17.30	− 1.731	0.086
Trial 5	21.84	18.25	− 0.983	0.336
Trial 6	22.00	18.10	− 1.068	0.296
Maximum force (N)				
Trial 1	21.25	19.75	− 0.406	0.698
Trial 2	20.10	20.90	− -0.216	0.841
Trial 3	17.85	23.15	− 1.434	0.157
Trial 4	21.60	19.40	− 0.595	0.565
Trial 5	17.84	22.05	− 1.152	0.258
Trial 6	18.58	21.35	− 0.759	0.461
Mean non-zero force (N)				
Trial 1	21.53	19.48	− 0.555	0.583
Trial 2	19.23	21.78	− 0.690	0.495
Trial 3	17.55	23.45	− 1.596	0.114
Trial 4	20.58	20.43	− 0.041	0.968
Trial 5	17.16	21.84	− 1.299	0.201
Trial 6	17.95	21.05	− 0.861	0.402
Maximum impulse (Ns)				
Trial 1	23.30	17.70	− -1.515	0.134
Trial 2	20.95	20.05	− 0.243	0.820
Trial 3	20.18	20.83	− 0.176	0.862
Trial 4	21.00	20.00	− 0.271	0.799
Trial 5	18.84	21.10	− 0.618	0.550
Trial 6	21.26	18.80	− 0.674	0.513
Force volume (N^3^)				
Trial 1	22.20	18.80	− 0.920	0.369
Trial 2	20.08	20.93	− 0.230	0.820
Trial 3	18.20	22.80	− 1.244	0.202
Trial 4	20.53	20.48	− 0.014	0.989
Trial 5	17.84	22.05	− 1.152	0.258
Trial 6	19.13	20.83	− 0.464	0.647

Bold indicate an outcome with a *p* <0.05 was regarded as statistically significant

### Construct validation using linear regression analyses

The regression analysis indicate clear regression of the total task time (*p* ≤ 0.05) and partial regression for the maximum impulse (Trial 1 *p* ≤ 0.001, Trial 2 *p* = 0.050 and Trial 6 *p* = 0.007), maximum force (Trial 1 *p* = 0.029), force volume (Trial 1 *p* = 0.006 and Trial 2 *p* = 0.029) and mean non-zero force (Trial 1 *p* = 0.023) (Supplemental file C, Table C1–C5).

### Learning curve analysis

Comparing the first and sixth trial, all groups were able to complete the trials faster: novices (164 s vs. 97 s *p* = *0.011*), intermediates (103 s vs. 89 s *p* = *0.011*) and experts (106 s vs. 72 s *p* = *0.002*) (Table 3). Moreover, a decrease in the mean non-zero force was observed in the novice group (1.38 N vs. 1.22 N *p* = *0.040*) and expert group (1.07 N vs. 1.00 N *p* = *0.017*). Similarly, a decrease in max impulse was found in the novice group (27.01 Ns vs. 15.09 Ns *p* = *0.016*) and expert group (14.74 Ns vs. 10.45 Ns *p* = *0.017*). Lastly, the expert group were able to decrease the force volume (0.98 N^3^ vs. 0.66 N^3^ *p* = *0.019*).

**Table 3:** Robotic suturing trials medians and Wilcoxon signed-rank test of the novices, intermediates and experts

Trials	Trial 1 median	Trial 6 median	*Z* value	Asymp. Sig. (2 tailed)
Total time (s)				
Novice	164.3 s	96.62 s	− 2.535	**0.011**
Intermediate	102.9 s	88.80 s	− 2.539	**0.011**
Expert	105.6 s	72.39 s	− 3.103	**0.002**
Maximum force (N)				
Novice	6.32 N	5.05 N	− 1.69	0.091
Intermediate	5.48 N	5.83 N	− 0.485	0.627
Expert	4.40 N	4.14 N	− 1.138	0.255
Mean non-zero force (N)				
Novice	1.38 N	1.22 N	− 2.053	**0.040**
Intermediate	1.20 N	1.34 N	− 0.221	0.825
Expert	1.07 N	1.00 N	− 2.379	**0.017**
Maximum impulse (Ns)				
Novice	27.01 Ns	15.09 Ns	− 2.415	**0.016**
Intermediate	19.44 Ns	16.75 Ns	− 1.232	0.218
Expert	14.74 Ns	10.45 Ns	− 2.379	**0.017**
Force volume (N^3^)				
Novice	2.06 N^3^	1.63 N^3^	− 1.891	0.059
Intermediate	1.40 N^3^	1.88 N^3^	− 0.56	0.575
Expert	0.98 N^3^	0.66 N^3^	− 2.343	**0.019**

Bold indicate an outcome with a *p* <0.05 was regarded as statistically significant

## Discussion

This study showed construct validity evidence as the objective measuring system was able to show clear differences on objective force parameters and distinguish between novices, intermediates and experts on the da Vinci Surgical System. These difference are in line with our prior findings for laparoscopy [5, 7]. The system is not exclusively able to distinguish novices and experts but also intermediates and experts. The experts showed lower completion time and force parameter outcomes in almost all trials for all parameters.

Furthermore, all three groups improved in the completion time when comparing the first and last trial. The novices and experts improved in mean non-zero force, maximum impulse and force volume. This improvement of skill, based on objective measurements, and the learning curve in robotic tissue handling skill confirms and extends our prior findings [5–7, 16]. This is of interest in daily practice regarding robotic suturing and knot tying, since increased psychomotor skills and hand–eye coordination translate into better tissue manipulation skills [17, 18].

Previously, the importance of forces and tissue manipulation in different experience groups was described by our research team [5, 7]. For robotic surgery, the differences are even more divergent due to the difficult learning curve of tissue treatment in RAS due to the lack of haptic feedback. Furthermore, a significant higher number of suture breakage was observed in the novice and intermediate group. In terms of clinical relevance, differences in force measurements could potentially be indicative of differences in surgical performance or outcomes. For example, if one group of trainees consistently applies higher or lower forces during training, this could potentially translate to differences in surgical precision or the risk of complications during actual surgery.

The ability to plot learning curves allows us to use regression analysis combined with artificial intelligence and machine learning to create predictive software that can be used to provide custom training to the user including an estimation of the amount of time needed on the trainers. This can have a tremendous effect on cost related factors in a time that most education budgets are under pressure.

Although direct force feedback lacks in most robotic platforms and therefore ex vivo training is of paramount importance, this study is the first to analyze and report objective interaction force assessment in robotic surgery training [19]. Some studies have researched grip force but not the force exerted on the tissue [20]. Furthermore, studies have been conducted with excessive force and instrument collisions in the da Vinci Skills Simulator (dVSS) [21–26]. However, this is limited to a virtual reality environment and does not translate to force exerted on tissue by instruments.

A strength of this study is the large sample size of participants from two international academic hospitals and thus increasing the generalizability of the construct validation. The participants performed a standardized suture and knot tying task which can not only be applied in a broad spectrum of daily practice but also is representative for the assessment and validation of laparoscopic and general surgical skill [27, 28]. Another strength is the use of previously validated objective force and time and metrics that represent instrument and tissue handling skills in laparoscopic skills training [5, 7, 29]. Furthermore, all trials were recorded and participants, peers and supervisors can review the performances. A limitation is that no motion parameters could be measured, due to the discrepancies of trocar instrument and da Vinci instrument diameter. In future studies that include combined force, motion and time parameters, the diameter of the motion sensors should be adjusted.

## Conclusion

This study showed that it is possible to distinguish between different skill levels in robot-assisted surgery. This provides validity evidence and relevance for using objective assessment of tissue handling skills during simulation training for robotic surgery. Moreover, repetitive assessment showed learning curves over time. To ensure and quantify technical competency at the end of training, we advise surgical trainers to incorporate force-based assessment in robotic surgery training systems.

## Supplementary Information

Below is the link to the electronic supplementary material.

Supplementary file1 (DOCX 1786 kb)
