# Supplementary material for: Force-based assessment of tissue handling skills in simulation training for robot-assisted surgery
Source: Surg Endosc. 2023 Feb 9;37(6):4414–20. doi: 10.1007/s00464-023-09905-y (PMC10234900; doi:10.1007/s00464-023-09905-y)
Supplement: Supplementary file 1 — Supplementary file1 (DOCX 1786 kb) [file 464_2023_9905_MOESM1_ESM.docx]

**Supplemental file A**

**Fig A1**

*Robotic setup with the da Vinci Si Surgical System (Intuitive Surgical Inc., Sunnyvale, California USA).***
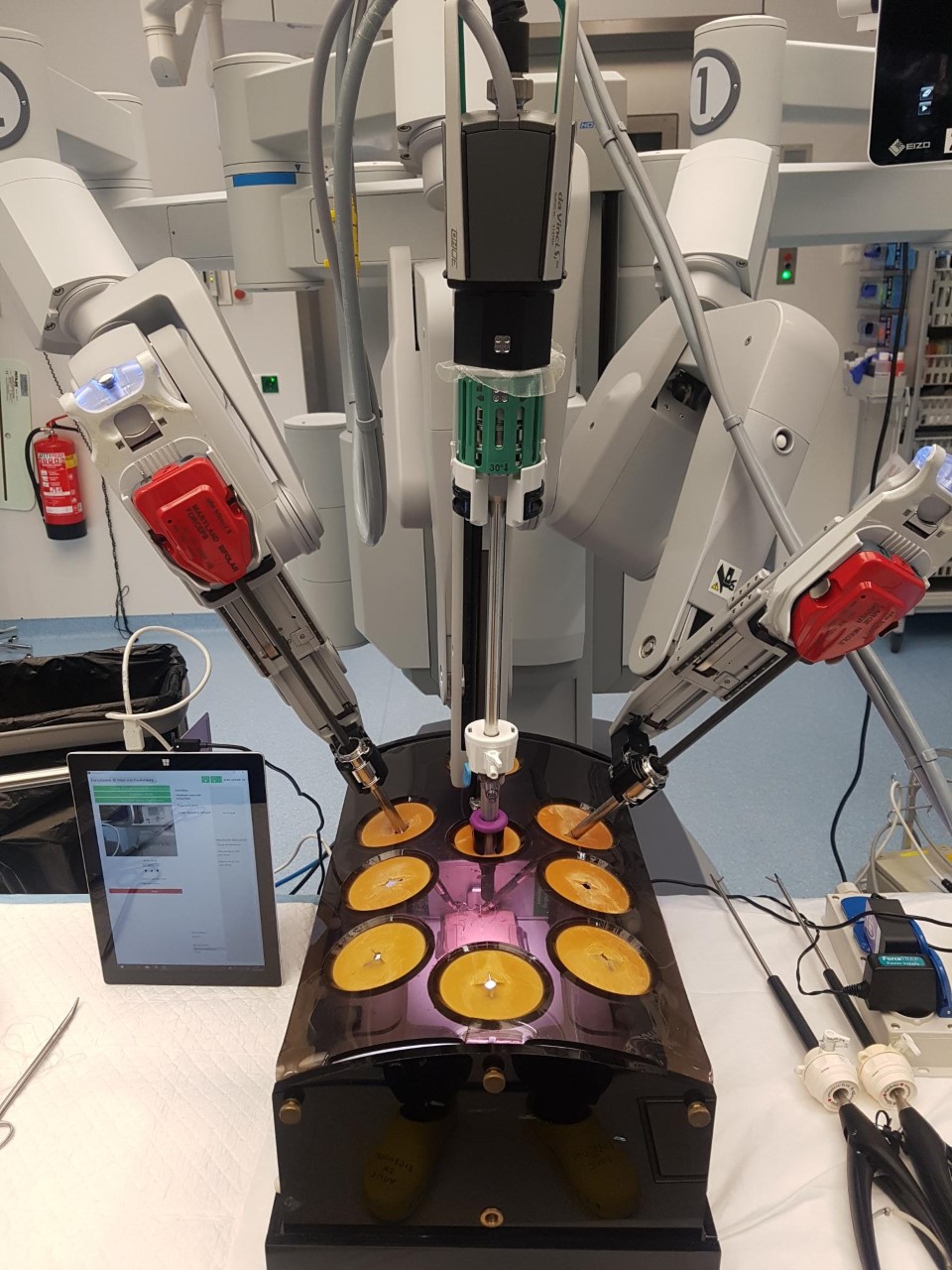
**

**Fig A2**

**
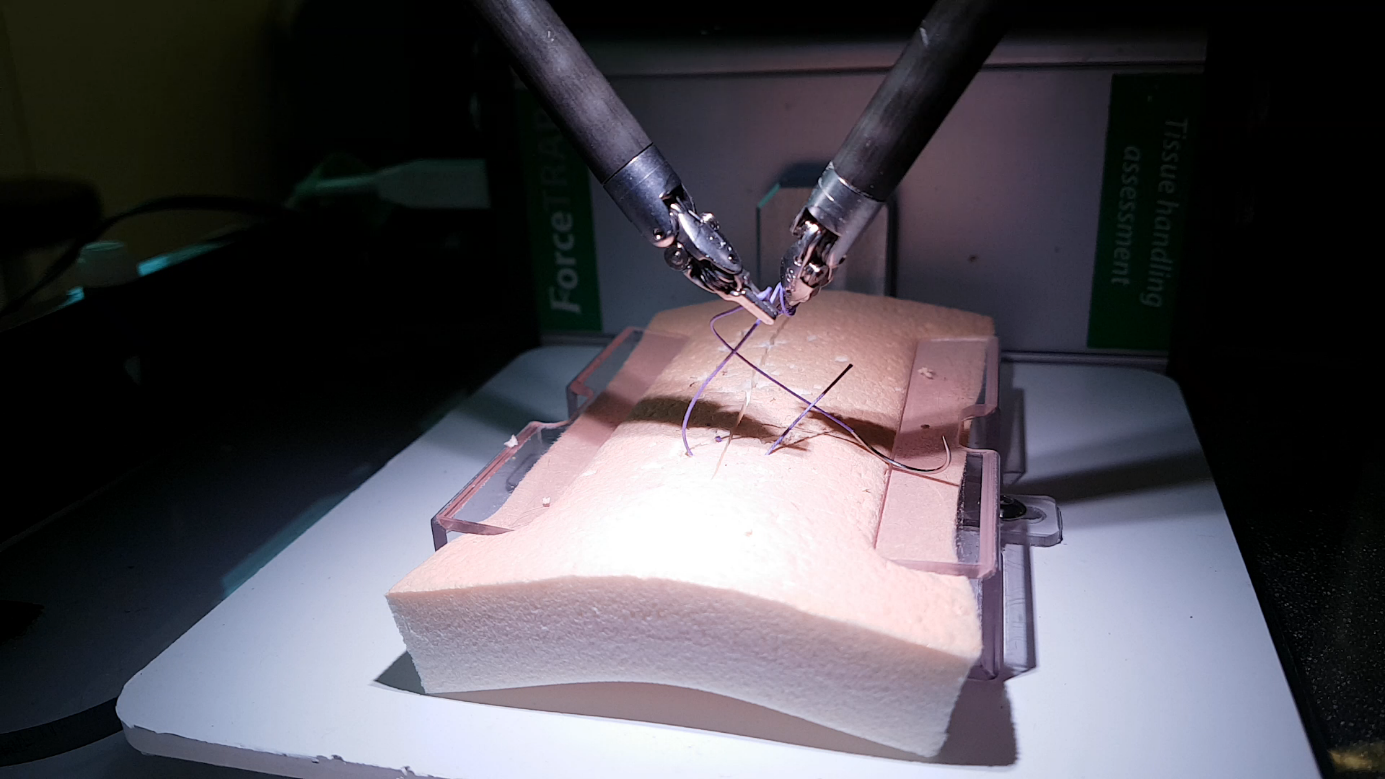
**
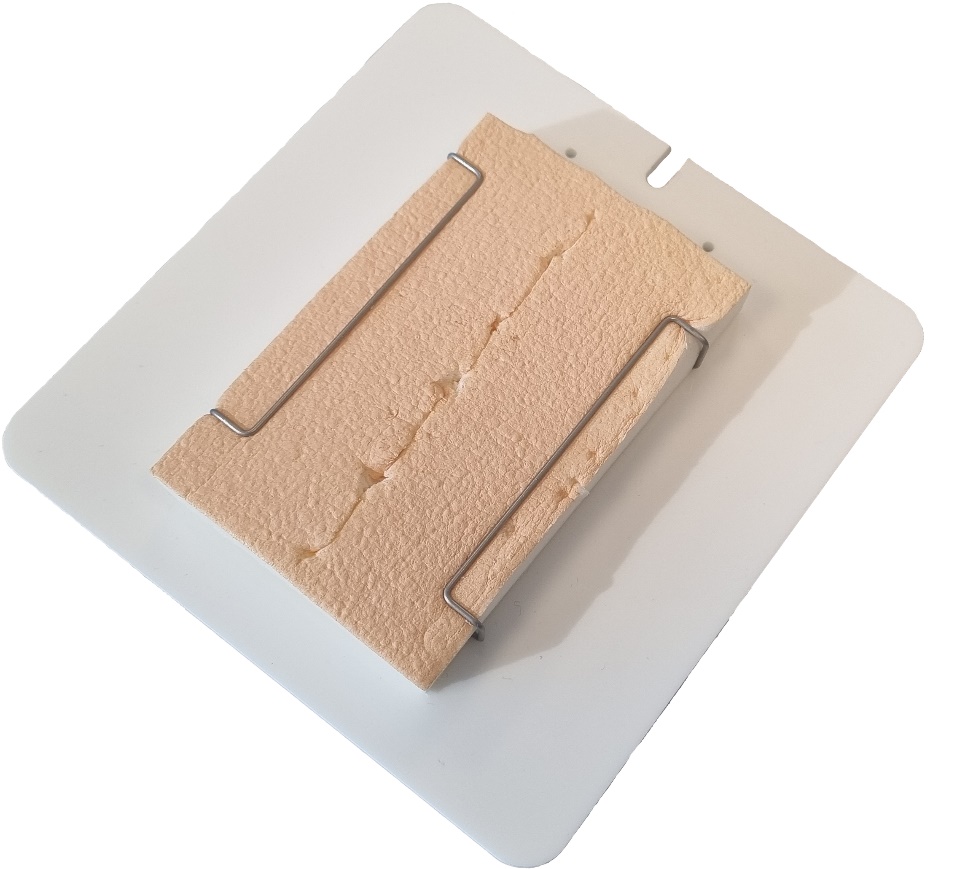
Suture pad used in the Robotic suture tasks

**Supplemental file B**

**Analyses for construct validation**

Overview of the Kruskal-Wallis tests and Mann-Whitney U tests:

**Table B1.**

*Robotic suturing task median and Kruskal-Wallis test of the novices. intermediates and experts.*

|  | Novice (n=20) | Intermediate  (n=20) | Expert  (n=20) | *p* |
| --- | --- | --- | --- | --- |
| Total time (s) |  |  |  |  |
| Trial 1 | 40.15 | 26.65 | 24.70 | **0.010** |
| Trial 2 | 37.90 | 28.75 | 24.85 | 0.053 |
| Trial 3 | 40.85 | 28.55 | 22.10 | **0.003** |
| Trial 4 | 38.15 | 29.45 | 22.00 | **0.013** |
| Trial 5 | 35.32 | 29.85 | 23.32 | 0.090 |
| Trial 6 | 33.79 | 28.75 | 20.19 | **0.042** |
|  |  |  |  |  |
| Maximum force (N) |  |  |  |  |
| Trial 1 | 35.20 | 32.65 | 23.65 | 0.089 |
| Trial 2 | 33.55 | 34.18 | 23.78 | 0.107 |
| Trial 3 | 28.43 | 36.40 | 26.68 | 0.172 |
| Trial 4 | 32.58 | 29.80 | 27.50 | 0.652 |
| Trial 5 | 27.95 | 34.10 | 26.21 | 0.306 |
| Trial 6 | 28.37 | 32.30 | 22.19 | 0.169 |
|  |  |  |  |  |
| Mean non-zero force (N) |  |  |  |  |
| Trial 1 | 35.28 | 32.33 | 23.90 | 0.102 |
| Trial 2 | 31.43 | 34.83 | 25.25 | 0.213 |
| Trial 3 | 26.60 | 36.33 | 28.58 | 0.177 |
| Trial 4 | 32.00 | 31.30 | 26.53 | 0.559 |
| Trial 5 | 26.03 | 34.11 | 26.87 | 0.256 |
| Trial 6 | 29.08 | 32.89 | 19.22 | **0.032** |
|  |  |  |  |  |
| Maximum impulse (Ns) |  |  |  |  |
| Trial 1 | 39.95 | 31.45 | 20.10 | **0.001** |
| Trial 2 | 34.50 | 32.25 | 24.75 | 0.181 |
| Trial 3 | 31.38 | 31.93 | 28.20 | 0.767 |
| Trial 4 | 35.00 | 33.00 | 21.58 | **0.032** |
| Trial 5 | 29.68 | 33.10 | 25.53 | 0.375 |
| Trial 6 | 34.11 | 29.45 | 18.94 | **0.018** |
|  |  |  |  |  |
| Force volume (N^3^) |  |  |  |  |
| Trial 1 | 37.55 | 31.85 | 22.10 | **0.018** |
| Trial 2 | 34.35 | 35.05 | 22.10 | **0.031** |
| Trial 3 | 28.88 | 35.98 | 26.65 | 0.211 |
| Trial 4 | 32.73 | 32.05 | 24.97 | 0.299 |
| Trial 5 | 28.42 | 34.75 | 25.05 | 0.189 |
| Trial 6 | 30.84 | 32.85 | 20.76 | 0.060 |
|  |  |  |  |  |

**Table B2.**

*Comparison between the novices and intermediates for the robotic suturing task. The Mann-Whitney U test was performed to determine any significant differences between the groups.*

|  | Novice (n=20) | Intermediate  (n=20) | Z-value | *p* |
| --- | --- | --- | --- | --- |
| Total time (s) |  |  |  |  |
| Trial 1 | 25.10 | 15.90 | -2.489 | **0.012** |
| Trial 2 | 23.70 | 17.30 | -1.731 | 0.086 |
| Trial 3 | 24.65 | 16.35 | -2.245 | **0.024** |
| Trial 4 | 23.70 | 17.30 | -1.731 | 0.086 |
| Trial 5 | 21.84 | 18.25 | -0.983 | 0.336 |
| Trial 6 | 22.00 | 18.10 | -1.068 | 0.296 |
|  |  |  |  |  |
| Maximum force (N) |  |  |  |  |
| Trial 1 | 21.25 | 19.75 | -0.406 | 0.698 |
| Trial 2 | 20.10 | 20.90 | -0.216 | 0.841 |
| Trial 3 | 17.85 | 23.15 | -1.434 | 0.157 |
| Trial 4 | 21.60 | 19.40 | -0.595 | 0.565 |
| Trial 5 | 17.84 | 22.05 | -1.152 | 0.258 |
| Trial 6 | 18.58 | 21.35 | -0.759 | 0.461 |
|  |  |  |  |  |
| Mean non-zero force (N) |  |  |  |  |
| Trial 1 | 21.53 | 19.48 | -0.555 | 0.583 |
| Trial 2 | 19.23 | 21.78 | -0.690 | 0.495 |
| Trial 3 | 17.55 | 23.45 | -1.596 | 0.114 |
| Trial 4 | 20.58 | 20.43 | -0.041 | 0.968 |
| Trial 5 | 17.16 | 21.84 | -1.299 | 0.201 |
| Trial 6 | 17.95 | 21.05 | -0.861 | 0.402 |
|  |  |  |  |  |
| Maximum impulse (Ns) |  |  |  |  |
| Trial 1 | 23.30 | 17.70 | -1.515 | 0.134 |
| Trial 2 | 20.95 | 20.05 | -0.243 | 0.820 |
| Trial 3 | 20.18 | 20.83 | -0.176 | 0.862 |
| Trial 4 | 21.00 | 20.00 | -0.271 | 0.799 |
| Trial 5 | 18.84 | 21.10 | -0.618 | 0.550 |
| Trial 6 | 21.26 | 18.80 | -0.674 | 0.513 |
|  |  |  |  |  |
| Force volume (N^3^) |  |  |  |  |
| Trial 1 | 22.20 | 18.80 | -0.920 | 0.369 |
| Trial 2 | 20.08 | 20.93 | -0.230 | 0.820 |
| Trial 3 | 18.20 | 22.80 | -1.244 | 0.202 |
| Trial 4 | 20.53 | 20.48 | -0.014 | 0.989 |
| Trial 5 | 17.84 | 22.05 | -1.152 | 0.258 |
| Trial 6 | 19.13 | 20.83 | -0.464 | 0.647 |
|  |  |  |  |  |

**Table B3.**

*Comparison between the novices and experts for the robotic suturing task. The Mann-Whitney U test was performed to determine any significant differences between the groups.*

|  | Novice (n=20) | Expert  (n=20) | Z-value | *p* |
| --- | --- | --- | --- | --- |
| Total time (s) |  |  |  |  |
| Trial 1 | 25.55 | 15.45 | -2.732 | **0.006** |
| Trial 2 | 24.70 | 16.30 | -2.272 | **0.023** |
| Trial 3 | 26.70 | 14.30 | -3.354 | **0.001** |
| Trial 4 | 24.95 | 14.79 | -2.782 | **0.005** |
| Trial 5 | 23.47 | 15.53 | -2.204 | **0.027** |
| Trial 6 | 21.79 | 13.50 | -2.384 | **0.017** |
|  |  |  |  |  |
| Maximum force (N) |  |  |  |  |
| Trial 1 | 24.45 | 16.55 | -2.137 | **0.033** |
| Trial 2 | 23.95 | 17.05 | -1.867 | 0.063 |
| Trial 3 | 21.08 | 19.93 | -0.311 | 0.758 |
| Trial 4 | 21.48 | 18.45 | -0.829 | 0.411 |
| Trial 5 | 20.11 | 18.89 | -0.336 | 0.751 |
| Trial 6 | 19.79 | 15.88 | -1.126 | 0.271 |
|  |  |  |  |  |
| Mean non-zero force (N) |  |  |  |  |
| Trial 1 | 24.25 | 16.75 | -2.030 | **0.043** |
| Trial 2 | 22.70 | 18.30 | -1.190 | 0.242 |
| Trial 3 | 19.55 | 21.45 | -0.514 | 0.620 |
| Trial 4 | 21.93 | 17.97 | -1.082 | 0.283 |
| Trial 5 | 18.87 | 20.13 | -0.350 | 0.729 |
| Trial 6 | 21.13 | 14.28 | -1.972 | **0.048** |
|  |  |  |  |  |
| Maximum impulse (Ns) |  |  |  |  |
| Trial 1 | 27.15 | 13.85 | -3.598 | **≤0.001** |
| Trial 2 | 24.05 | 16.95 | -1.921 | 0.056 |
| Trial 3 | 21.70 | 19.30 | -0.649 | 0.529 |
| Trial 4 | 24.50 | 15.26 | -2.529 | **0.011** |
| Trial 5 | 20.84 | 18.16 | -0.744 | 0.470 |
| Trial 6 | 22.84 | 12.25 | -3.046 | **0.002** |
|  |  |  |  |  |
| Force volume (N^3^) |  |  |  |  |
| Trial 1 | 25.85 | 15.15 | -2.894 | **0.003** |
| Trial 2 | 24.78 | 16.23 | -2.313 | **0.020** |
| Trial 3 | 21.18 | 19.83 | -0.365 | 0.718 |
| Trial 4 | 22.70 | 17.16 | -1.517 | 0.134 |
| Trial 5 | 20.58 | 18.42 | -0.599 | 0.563 |
| Trial 6 | 21.71 | 14.91 | -1.933 | 0.052 |
|  |  |  |  |  |

**Table B4.**

*Comparison between the intermediates and experts for the robotic suturing task. The Mann-Whitney U test was performed to determine any significant differences between the groups.*

|  | Intermediates (n=20) | Expert  (n=20) | Z-value | *p* |
| --- | --- | --- | --- | --- |
| Total time (s) |  |  |  |  |
| Trial 1 | 21.25 | 19.75 | -0.406 | 0.698 |
| Trial 2 | 21.95 | 19.05 | -0.784 | 0.445 |
| Trial 3 | 22.70 | 18.30 | -1.190 | 0.242 |
| Trial 4 | 22.65 | 17.21 | -1.489 | 0.141 |
| Trial 5 | 22.10 | 17.79 | -1.180 | 0.247 |
| Trial 6 | 21.15 | 15.19 | -1.687 | 0.095 |
|  |  |  |  |  |
| Maximum force (N) |  |  |  |  |
| Trial 1 | 23.40 | 17.60 | -1.569 | 0.121 |
| Trial 2 | 23.78 | 17.23 | -1.772 | 0.076 |
| Trial 3 | 23.75 | 17.25 | -1.758 | 0.081 |
| Trial 4 | 20.90 | 19.05 | -0.506 | 0.627 |
| Trial 5 | 22.55 | 17.32 | -1.433 | 0.158 |
| Trial 6 | 21.45 | 14.81 | -1.879 | 0.062 |
|  |  |  |  |  |
| Mean non-zero force (N) |  |  |  |  |
| Trial 1 | 23.35 | 17.65 | -1.542 | 0.127 |
| Trial 2 | 23.55 | 17.45 | -1.650 | 0.102 |
| Trial 3 | 23.38 | 17.63 | -1.556 | 0.121 |
| Trial 4 | 21.38 | 18.55 | -0.773 | 0.444 |
| Trial 5 | 22.26 | 16.74 | -1.533 | 0.130 |
| Trial 6 | 21.84 | 13.44 | -2.418 | **0.015** |
|  |  |  |  |  |
| Maximum impulse (Ns) |  |  |  |  |
| Trial 1 | 24.25 | 16.75 | -2.029 | **0.043** |
| Trial 2 | 22.70 | 18.30 | -1.190 | 0.242 |
| Trial 3 | 21.60 | 19.40 | -0.595 | 0.565 |
| Trial 4 | 23.50 | 16.32 | -1.967 | **0.050** |
| Trial 5 | 22.50 | 17.37 | -1.405 | 0.166 |
| Trial 6 | 21.15 | 15.19 | -1.687 | 0.095 |
|  |  |  |  |  |
| Force volume (N^3^) |  |  |  |  |
| Trial 1 | 23.55 | 17.45 | -1.650 | 0.102 |
| Trial 2 | 24.63 | 16.38 | -2.232 | **0.024** |
| Trial 3 | 23.68 | 17.33 | -1.718 | 0.086 |
| Trial 4 | 22.08 | 17.82 | -1.166 | 0.247 |
| Trial 5 | 23.20 | 16.63 | -1.798 | 0.074 |
| Trial 6 | 22.53 | 14.85 | -2.149 | **0.030** |
|  |  |  |  |  |

**Supplemental file C**

**Analyses for construct validation**

Overview of the linear regression analyses:

**Table C1.**

*Robotic suture task time in seconds*

|  | Variable | B | 95% CI | β | t | *p* |
| --- | --- | --- | --- | --- | --- | --- |
| Trial 1 | (Constant) | 5.088 | [4.88; 5.30] |  | 48.608 | 0.000 |
|  | Experience | -0.260 | [-0.42; -0.10] | -0.388 | -3.203 | **0.002** |
|  |  |  |  |  |  |  |
| Trial 2 | (Constant) | 5.042 | [4.81; 5.27] |  | 44.269 | 0.000 |
|  | Experience | -0.290 | [-0.47; -0.11] | -0.396 | -3.285 | **0.002** |
|  |  |  |  |  |  |  |
| Trial 3 | (Constant) | 4.915 | [4.71; 5.12] |  | 47.945 | 0.000 |
|  | Experience | -0.306 | [-0.47; -0.15] | -0.451 | -3.849 | **≤0.001** |
|  |  |  |  |  |  |  |
| Trial 4 | (Constant) | 4.835 | [4.63; 5.04] |  | 47.087 | 0.000 |
|  | Experience | -0.300 | [-0.46; -0.14] | -0.442 | -3.722 | **≤0.001** |
|  |  |  |  |  |  |  |
| Trial 5 | (Constant) | 4.725 | [98.30; 159.00] |  | 41.071 | 0.000 |
|  | Experience | -0.223 | [-63.90; 1.34] | -0.316 | -2.491 | **0.016** |
|  |  |  |  |  |  |  |
| Trial 6 | (Constant) | 4.723 | [4.51; 4.93] |  | 45.050 | 0.000 |
|  | Experience | -0.287 | [-0.46; -0.12] | -0.422 | -3.387 | **0.001** |

**Table C2.**

*Robotic suture task maximum impulse in Ns*

|  | Variable | B | 95% CI  [LB; UB] | β | t | *p* |
| --- | --- | --- | --- | --- | --- | --- |
| Trial 1 | (Constant) | 3.494 | [3.23; 3.76] |  | 26.182 | 0.000 |
|  | Experience | -0.384 | [-0.59; -0.18] | -0.438 | -3.714 | **≤0.001** |
|  |  |  |  |  |  |  |
| Trial 2 | (Constant) | 3.210 | [2.908 3.512] |  | 21.278 | 0.000 |
|  | Experience | -0.234 | [-0.47; 0.01] | -0.254 | -1.999 | **0.050** |
|  |  |  |  |  |  |  |
| Trial 3 | (Constant) | 0.495 | [-0.13; 1.12] |  | 1.577 | 0.120 |
|  | Experience | -0.111 | [-0.60; 0.38] | -0.060 | -0.458 | 0.649 |
|  |  |  |  |  |  |  |
| Trial 4 | (Constant) | 0.461 | [-0.10; 1.02] |  | 1.640 | 0.106 |
|  | Experience | -0.180 | [-0.62; 0.26] | -0.108 | -0.816 | 0.418 |
|  |  |  |  |  |  |  |
| Trial 5 | (Constant) | 2.913 | [2.61; 3.21] |  | 19.482 | 0.000 |
|  | Experience | -0.136 | [-0.37; 0.10] | -0.154 | -1.170 | 0.247 |
|  |  |  |  |  |  |  |
| Trial 6 | (Constant) | 3.045 | [2.74; 3.35] |  | 20.237 | 0.000 |
|  | Experience | -0.342 | [-0.59; -0.10] | -0.360 | -2.812 | **0.007** |

**Table C3.**

*Robotic suture task maximum absolute force in Newton*

|  | Variable | B | 95% CI | β | t | *p* |
| --- | --- | --- | --- | --- | --- | --- |
| Trial 1 | (Constant) | 1.927 | [1.69; 2.16] |  | 16.324 | 0.000 |
|  | Experience | -0.205 | [-0.39; -0.02] | -0.283 | -2.246 | **0.029** |
|  |  |  |  |  |  |  |
| Trial 2 | (Constant) | 1.838 | [1.54; 2.13] |  | 12.509 | 0.000 |
|  | Experience | -0.150 | [-0.38; 0.08] | -0.170 | -1.315 | 0.194 |
|  |  |  |  |  |  |  |
| Trial 3 | (Constant) | 1.711 | [1.44; 1.99] |  | 12.464 | 0.000 |
|  | Experience | -0.019 | [-0.23; 0.19] | -0.024 | -0.181 | 0.857 |
|  |  |  |  |  |  |  |
| Trial 4 | (Constant) | 1.651 | [1.39; 1.91] |  | 12.786 | 0.000 |
|  | Experience | -0.044 | [-0.25; 0.16] | -0.058 | -0.438 | 0.663 |
|  |  |  |  |  |  |  |
| Trial 5 | (Constant) | 1.578 | [1.35; 1.81] |  | 13.566 | 0.000 |
|  | Experience | -0.009 | [-0.19; 0.17] | -0.013 | -0.096 | 0.924 |
|  |  |  |  |  |  |  |
| Trial 6 | (Constant) | 1.669 | [1.46; 1.88] |  | 15.759 | 0.000 |
|  | Experience | -0.062 | [-0.23; 0.11] | -0.100 | -0.729 | 0.469 |

**Table C4.**

*Robotic suture task force volume in N*^3^

|  | Variable | B | 95% CI | β | t | *p* |
| --- | --- | --- | --- | --- | --- | --- |
| Trial 1 | (Constant) | 1.086 | [0.54; 1.63] |  | 4.000 | 0.000 |
|  | Experience | -0.599 | [-1.02; -0.18] | -0.350 | -2.846 | **0.006** |
|  |  |  |  |  |  |  |
| Trial 2 | (Constant) | 0.825 | [0.28; 1.37] |  | 3.012 | 0.004 |
|  | Experience | -0.474 | [-0.90; -0.05] | -0.281 | -2.233 | **0.029** |
|  |  |  |  |  |  |  |
| Trial 3 | (Constant) | 0.495 | [-0.13; 1.12] |  | 1.577 | 0.120 |
|  | Experience | -0.111 | [-0.60; 0.38] | -0.060 | -0.458 | 0.649 |
|  |  |  |  |  |  |  |
| Trial 4 | (Constant) | 0.461 | [-0.10; 1.02] |  | 1.640 | 0.106 |
|  | Experience | -0.180 | [-0.62; 0.26] | -0.108 | -0.816 | 0.418 |
|  |  |  |  |  |  |  |
| Trial 5 | (Constant) | 0.313 | [-0.26; 0.89] |  | 1.085 | 0.282 |
|  | Experience | -0.136 | [-0.58; 0.31] | -0.081 | -0.607 | 0.547 |
|  |  |  |  |  |  |  |
| Trial 6 | (Constant) | 0.479 | [-0.02; 0.98] |  | 1.917 | 0.060 |
|  | Experience | -0.334 | [-0.73; 0.07] | -0.222 | -1.676 | 0.100 |

**Table C5.**

*Robotic suture task mean non-zero force in Newton*

|  | Variable | B | 95% CI | β | t | *p* |
| --- | --- | --- | --- | --- | --- | --- |
| Trial 1 | (Constant) | 0.393 | [0.23; 0.56] |  | 4.797 | 0.000 |
|  | Experience | -0.148 | [-0.28; -0.02] | -0.293 | -2.331 | **0.023** |
|  |  |  |  |  |  |  |
| Trial 2 | (Constant) | 0.271 | [0.12; 0.43] |  | 3.483 | 0.001 |
|  | Experience | -0.091 | [-0.21; 0.03] | -0.195 | -1.516 | 0.135 |
|  |  |  |  |  |  |  |
| Trial 3 | (Constant) | 0.227 | [0.04; 0.42] |  | 2.404 | 0.019 |
|  | Experience | 0.007 | [-0.14; 0.15] | 0.013 | 0.097 | 0.923 |
|  |  |  |  |  |  |  |
| Trial 4 | (Constant) | 0.213 | [0.05; 0.38] |  | 2.555 | 0.013 |
|  | Experience | -0.056 | [-0.19; 0.08] | -0.113 | -0.862 | 0.392 |
|  |  |  |  |  |  |  |
| Trial 5 | (Constant) | 0.200 | [0.04; 0.36] |  | 2.574 | 0.013 |
|  | Experience | -0.006 | [-0.13; 0.12] | -0.014 | -0.101 | 0.920 |
|  |  |  |  |  |  |  |
| Trial 6 | (Constant) | 0.261 | [0.12; 0.40] |  | 3.699 | 0.001 |
|  | Experience | -0.095 | [-0.21; 0.02] | -0.225 | -1.662 | 0.103 |

**Supplemental file D**

**Analyses for skills progression**

Overview of Wilcoxon signed-rank tests:

**Table D1.**

*Robotic suturing trials medians and Wilcoxon signed-rank test of the novices, intermediates and experts.*

| Trials | Trial 1  Median | Trial 6  Median | Z value | Asymp. Sig. (2 tailed) |
| --- | --- | --- | --- | --- |
| Total time (s) |  |  |  |  |
| Novice | 164.3 s | 96.62 s | -2.535 | **0.011** |
| Intermediate | 102.9 s | 88.80 s | -2.539 | **0.011** |
| Expert | 105.6 s | 72.39 s | -3.103 | **0.002** |
| Maximum force (N) |  |  |  |  |
| Novice | 6.32 N | 5.05 N | -1.69 | 0.091 |
| Intermediate | 5.48 N | 5.83 N | -0.485 | 0.627 |
| Expert | 4.40 N | 4.14 N | -1.138 | 0.255 |
| Mean non-zero force (N) |  |  |  |  |
| Novice | 1.38 N | 1.22 N | -2.053 | **0.040** |
| Intermediate | 1.20 N | 1.34 N | -0.221 | 0.825 |
| Expert | 1.07 N | 1.00 N | -2.379 | **0.017** |
| Maximum impulse (Ns) |  |  |  |  |
| Novice | 27.01 Ns | 15.09 Ns | -2.415 | **0.016** |
| Intermediate | 19.44 Ns | 16.75 Ns | -1.232 | 0.218 |
| Expert | 14.74 Ns | 10.45 Ns | -2.379 | **0.017** |
| Force volume (N^3^) |  |  |  |  |
| Novice | 2.06 N^3^ | 1.63 N^3^ | -1.891 | 0.059 |
| Intermediate | 1.40 N^3^ | 1.88 N^3^ | -0.56 | 0.575 |
| Expert | 0.98 N^3^ | 0.66 N^3^ | -2.343 | **0.019** |
|  |  |  |  |  |

**Supplemental File E**

Power analysis of the initial acquired data of the thirty participants of the Dept. of Surgery, Heidelberg University, Germany:

To check the initial results of Heidelberg, post hoc power analysis was performed to determine the effect of the group size on the outcomes. The calculations were based on the following conditions: power (1-β) of 0.80, two sided and a test significance level (α) of 0.05. This was performed for both the time and force outcomes of the robotic trials. The time analysis showed no increase in the amount of participants needed for statistical significance. However, the force calculations presented an required increase of the participants for each trial (trial 1 n=61 , trial 2 n=53, trial 3 n=55, trial 4 n=46, trial 5 n=196 and trial 6 n=36) to achieve statistical significance on the level of α = 0.05.
